# High resolution melting analysis for rapid and sensitive *EGFR *and *KRAS *mutation detection in formalin fixed paraffin embedded biopsies

**DOI:** 10.1186/1471-2407-8-142

**Published:** 2008-05-21

**Authors:** Hongdo Do, Michael Krypuy, Paul L Mitchell, Stephen B Fox, Alexander Dobrovic

**Affiliations:** 1 Molecular Pathology Research and Development Laboratory, Department of Pathology, Peter MacCallum Cancer Centre, Locked Bag 1, A'Beckett St, Melbourne, Victoria 8006, Australia; 2 Department of Pathology, University of Melbourne, Parkville, Victoria, 3010, Australia; 3 Ludwig Medical Oncology Department, Austin Hospital, Heidelberg, Victoria, 3084, Australia

## Abstract

**Background:**

Epithelial growth factor receptor (*EGFR*) and *KRAS *mutation status have been reported as predictive markers of tumour response to *EGFR *inhibitors. High resolution melting (HRM) analysis is an attractive screening method for the detection of both known and unknown mutations as it is rapid to set up and inexpensive to operate. However, up to now it has not been fully validated for clinical samples when formalin-fixed paraffin-embedded (FFPE) sections are the only material available for analysis as is often the case.

**Methods:**

We developed HRM assays, optimised for the analysis of FFPE tissues, to detect somatic mutations in *EGFR *exons 18 to 21. We performed HRM analysis for *EGFR *and *KRAS *on DNA isolated from a panel of 200 non-small cell lung cancer (NSCLC) samples derived from FFPE tissues.

**Results:**

All 73 samples that harboured *EGFR *mutations previously identified by sequencing were correctly identified by HRM, giving 100% sensitivity with 90% specificity. Twenty five samples were positive by HRM for *KRAS *exon 2 mutations. Sequencing of these 25 samples confirmed the presence of codon 12 or 13 mutations. *EGFR *and *KRAS *mutations were mutually exclusive.

**Conclusion:**

This is the first extensive validation of HRM on FFPE samples using the detection of *EGFR *exons 18 to 21 mutations and *KRAS *exon 2 mutations. Our results demonstrate the utility of HRM analysis for the detection of somatic *EGFR *and *KRAS *mutations in clinical samples and for screening of samples prior to sequencing. We estimate that by using HRM as a screening method, the number of sequencing reactions needed for *EGFR *and *KRAS *mutation detection can be reduced by up to 80% and thus result in substantial time and cost savings.

## Background

Lung cancer is the leading cause of cancer-related death, accounting for one third of all cancer mortality worldwide due to high incidence, advanced stage at diagnosis and aggressive tumour behaviour [1]. Non-small cell lung cancer (NSCLC), comprising 80% of all lung cancer cases, has a poor prognosis if diagnosed at an advanced stage. There is a low median survival of less than one year after diagnosis when treated by conventional chemotherapy [2].

The epidermal growth factor receptor (EGFR) is a member of the ErbB receptor tyrosine kinase family. EGFR has been found to be over-expressed in a variety of human malignancies [3,4]. Activation of EGFR results in the initiation of a diverse range of cellular signalling pathways, including cell proliferation and protection of the cell from apoptosis [5,6]. Activating mutations in the tyrosine kinase domain of the EGFR gene (*EGFR*) have been shown to be associated with a dramatic response to the tyrosine kinase inhibitors (TKIs), such as gefitinib and erlotinib [7-10]. These mutations are located in exons 18 to 21 and are more common in females, non-smokers, tumours with a histological diagnosis of adenocarcinoma, and individuals of Asian descent [11]. In cell line studies, these mutations have been shown to induce oncogenic transformation of fibroblasts and lung epithelial cells [7,12-15].

*KRAS *is a member of the Ras gene family which encode small G proteins with intrinsic GTPase activity. The KRAS protein plays a key role in Ras/MAPK signalling which is involved in multiple pathways including proliferation, differentiation and apoptosis [16]. *KRAS *mutations which are found in 33% of NSCLC are restricted to specific codons; more commonly codons 12 and 13 in exon 2 and rarely codons 59 and 61 in exon 3 [16,17]. These mutations alter the conformation of KRAS causing impaired GTPase activity resulting in the protein being constitutively active. *KRAS *mutation testing is an important adjunct to *EGFR *testing because *KRAS *mutations are significantly associated with absence of responsiveness to *EGFR *inhibitors and are mutually exclusive to *EGFR *mutations [18-20]. Thus, the mutational status of *EGFR *and *KRAS *can provide important information for stratification of NSCLC patients to receive molecularly targeted treatment with tyrosine kinase inhibitors.

Currently, the most widely used method for *EGFR *and *KRAS *mutation detection is direct sequencing. To be successful, the sequencing methodology requires a sufficient amount of tumour material of relatively good quality, which is difficult to obtain from cancer patients with inoperable tumours. In addition, the high cost, limited sensitivity and time consuming nature of sequencing has prompted the development of alternative methods that are more cost effective, faster, easier to perform, and more sensitive. The relatively low sensitivity of sequencing in somatic mutation detection [21] is a particular problem in lung cancer where biopsies are often small and often contain only a small proportion of neoplastic cells. Studies using denaturing high performance liquid chromatography (DHPLC) have found additional *EGFR *mutations, which were undetected by sequencing [22-24]. However DHPLC, requiring extra sample handling after PCR amplification and expensive instrumentation, is relatively slow as the samples can only be analysed sequentially.

High resolution melting (HRM) analysis is a recently developed methodology that has enormous potential for the detection of DNA sequence changes [25]. New instruments combined with DNA intercalating dyes that can be used at saturating concentrations allow the discrimination of sequence changes in PCR amplicons without manual handling of PCR products. The recent application of HRM to mutation scanning and SNP genotyping as well as DNA methylation studies have been demonstrated [26-31]. In particular, the HRM methodology has shown great promise for the detection of heterozygous germline mutations as well as somatic mutations e.g. in the *KIT*, *BRAF *and *TP53 *genes [26,32,33].

Formalin fixation followed by paraffin embedding of tissue specimens is a widely used preservation method because it helps to maintain morphological features of the tissue specimen [34]. However, irreversible damage to DNA can occur during this process or subsequent prolonged storage, resulting in an adverse effect on DNA quality [35].

As FFPE tissues are the most common clinical specimens used for detection of the *EGFR *and *KRAS *mutations, the validation of HRM using DNA samples extracted from FFPE tissues is essential for its application as a screening method for mutation detection in these genes. In this study, we developed HRM assays to evaluate the efficacy of this methodology for screening *EGFR *mutations in exons 18 to 21 using a panel of 200 NSCLC FFPE biopsies. *KRAS *exon 2 mutations at codon 12 and 13 were also screened in this sample set using a previously described HRM assay.

## Methods

### Samples

A total of 200 lung cancer biopsy specimens were analysed from specimens sent to the Peter MacCallum Cancer Centre, Melbourne, Australia for *EGFR *mutation detection by direct sequencing. These patients were often referred for *EGFR *mutation testing by clinicians since their clinical features and history were suggestive of those previously reported for patients with EGFR-associated mutations; histological diagnosis of adenocarcinoma, female gender, non-smoker status and Asian ethnicity. Of the 200 samples, 141 were adenocarcinomas, 24 were large cell carcinomas, 10 were squamous cell carcinomas, and 25 were of other or unknown histologies. The samples were independently assessed by pathologists from both the referring hospital, and from the Peter MacCallum Cancer Centre. Tumour-rich areas on a hematoxylin and eosin slide were marked by the pathologist to ensure that the maximum amount of tumour material was collected for the genetic testing. Written informed consent was obtained from all patients or families prior to testing. This study was approved by the Ethics of Human Research Committee at the Peter MacCallum Cancer Centre (project number 03/90).

### DNA extraction from formalin-fixed paraffin-embedded (FFPE) tissue

Tissue sections of 5 μm thickness were obtained from FFPE tissues and stained with Methyl green. The tumour-rich areas were micro-dissected using a 21G needle and the samples underwent proteinase K digestion in a rotating incubator at 56°C for 3 days. Genomic DNA was extracted using the DNeasy Tissue kit (Qiagen, Hilden, Germany) according to the manufacturer's protocol and was kept at 4°C before use.

### Design of HRM primers

Several factors were taken into account during the design of primers for this study. As FFPE DNA was used, primers that would yield reproducible amplification from degraded templates, or DNA in which the quality has been compromised were designed. As a result, primers giving rise to shorter amplicons were chosen as they are more likely to result in satisfactory amplification from degraded FFPE DNA. Furthermore, existing primer pairs that were used for the sequencing of *EGFR *exons 18 to 21 (Table 1) were found to be inappropriate for HRM as, with these longer amplicons (233, 294, 356 and 295 bp for exons 18 to 21 respectively), samples harbouring mutations were not efficiently distinguished from wild type samples in melting analysis. We thus chose to design primers that flanked the exons as closely as possible. As single nucleotide polymorphisms (SNPs) cannot be readily distinguished from mutations by HRM analysis, designing shorter amplicons in this way also minimises the inclusion of these confounding sequence variants. Most intronic SNPs can thus be excluded from the amplicons. If a SNP is close to the exon boundary, the primer can be placed over the SNP and a mismatched base with no allelic preference can be introduced at the SNP position [26]. Thus, the base 'A' was incorporated at the c.2184+19G>A position within the *EGFR *exon 18 reverse primer. The primers were designed to have annealing temperatures of around 60°C as predicted by Primer Express 1.5 software (Applied Biosystems, Foster City, CA). Each amplicon was analysed to ensure that it contained only a single melting domain using the Poland algorithm [36].

**Table 1:** *EGFR *HRM and sequencing primer sequences

**Exon**	**Primer name**	**Sequence**	**Amplicon size**
**Sequencing^#^**			
18	EGFR18_m13_F	5'-CATGGTGAGGGCTGAGGTGA-3'	233 bp
	EGFR18_m13_R	5'-CCCCACCAGACCATGAGAGG-3'	
19	EGFR19_m13_F	5'-GTGCATCGCTGGTAACATCCA-3'	294 bp
	EGFR19_m13_R	5'-GGAGATGAGCAGGGTCTAGAGCA-3'	
20	EGFR20_m13_F	5'-CGCATTCATGCGTCTTCACC-3'	356 bp
	EGFR20_m13_R	5'-CTATCCCAGGAGCGCAGACC-3'	
21	EGFR21_m13_F	5'-TGGCATGAACATGACCCTGAA-3'	295 bp
	EGFR21_m13_R	5'-CAGCCTGGTCCCTGGTGTC-3'	
**HRM**			
18	EGFR_ex18_F	5'-CATGGTGAGGGCTGAGGTGA-3'	199 bp
	EGFR_ex18_R	5'-CCAGAGG(A*)CTGTGCCAGGGAC-3'	
19	EGFR_ex19_F	5'-GTGCATCGCTGGTAACATCCA-3'	250 bp
	EGFR_ex19_R	5'-AAAGGTGGGCCTGAGGTTCA-3'	
20a	EGFR_ex20a_F	5'-AAGCCACACTGACGTGCCTCT-3'	121 bp
	EGFR_ex20a_R	5'-GCGTGATGAG(G*)TGCACGGT-3'	
20b	EGFR_ex20b_F	5'-CCTCCACCGTGCA(C*)CTCATC-3'	146 bp
	EGFR_ex20b_R	5'-CCCGTATCTCCCTTCCCTGA-3'	
21	EGFR_ex21_F	5'-CCTCACAGCAGGGTCTTCTCTG-3'	210 bp
	EGFR_ex21_R	5'-TGGCTGACCTAAAGCCACCTC-3'	

^# ^M13 sequences (not shown) are attached to all sequencing primers.

(*) indicates the position of the mismatched base which is introduced to prevent SNP interference and allele-specific PCR for the common SNPs c.2184+19G>A and c.2361G>T, in intron 18 and exon 20 respectively.

### HRM assays

The *EGFR *HRM primer sequences are shown in Table 1. *EGFR *exon 18, 19 and 21 HRM primers were designed to span the entire exon with product sizes of 199, 250 and 210 bp respectively. *EGFR *exon 20 was amplified in two fragments (ex20a and ex20b) with product sizes of 121 bp and 146 bp. The *KRAS *exon 2 primers which amplified a 92 bp product were described previously [37].

PCR for HRM analysis was performed in 0.1 ml tubes on the Rotor-Gene 6000™ (Corbett Research, Sydney, Australia) in the presence of the fluorescent DNA intercalating dye, SYTO 9 (Invitrogen, Carlsbad, CA). The reaction mixture in a 20 μl final volume contained; 1× PCR buffer, 2.5 mM MgCl_2_, 200–400 nM forward primer, 200–400 nM reverse primer, 5 ng of genomic DNA, 200 μM of dNTPs, 5 μM of SYTO 9, 0.5 U of HotStarTaq (Qiagen) polymerase and PCR grade water. The cycling and melting conditions for *EGFR *exons 18 to 21 were as follows; one cycle of 95°C for 15 min; 45–50 cycles of 95°C for 10 s, 65°C for 10 s with an initial 10 cycles of touchdown (1°C/cycle), 72°C for 30 s; one cycle of 97°C for 1 min and a melt from 70°C to 95°C rising 0.2°C per second. The cycling and melting conditions for *KRAS *exon 2 were as follows; one cycle of 95°C for 15 min; 50 cycles of 95°C for 10 s, 67.5°C for 5 s with an initial 10 cycles of touchdown (1°C/cycle), 72°C for 20 s; one cycle of 97°C for one min and a melt from 70°C to 95°C rising 0.2°C per second. The genomic DNA was diluted to 2.5 ng/μl (5 ng tested) to provide a consistent testing condition. All samples were tested in duplicate.

### HRM analysis

High resolution melting analysis was performed on the Rotor-Gene 6000 Software (v1.7) and analysed by two scientists who were blinded to the sequencing results. The normalised graph and the difference graph were used to analyse the data. The normalised graph was generated by the monitoring of dissociation of the fluorescent dye from double-stranded DNA as the temperature increased. The dye (SYTO 9) used in the current study can only fluoresce when it is intercalated into double-strand DNA. The normalised graph shows the degree of reduction in fluorescence over a temperature range (typically 70°C to 95°C). All samples including the wild-type were plotted according to their melting profiles. In the difference graph, the melting profiles of each sample were compared to that of the wild-type which was converted to a horizontal line. Significant deviations from the horizontal line (relative to the spread of the wild type controls) were indicative of sequence changes within the amplicon analysed. Samples with aberrant melting curves were recorded as HRM mutation positive. HRM results were compared with sequencing results for the validation of HRM analysis in the detection of *EGFR *mutations.

### DNA sequencing

All samples were sequenced for the detection of *EGFR *mutations in exons 18 to 21 using M13 tagged primers (Table 1). The reaction mixture in a total of 25 μl contained the following; 1× PCR buffer, 2.5 mM MgCl_2_, 200 nM of each primer, 50 ng of genomic DNA (if possible), 200 μM of dNTPs, 0.5 U of HotStarTaq polymerase and PCR grade water. The PCR reaction was performed using the following conditions; initial denaturation at 95°C for 15 min; 40 cycles of 94°C for 45 s, 65°C for 45 s, 72°C for 45 s; one cycle of 72°C for 10 min. 6 μl of the PCR products were purified with ExoSapIT (GE Healthcare, Little Chalfont, England) followed by a sequencing reaction with Big Dye Terminator v3.1 (Applied Biosystems, Foster City, CA) according to the manufacturer's protocol. The sequencing products were ethanol precipitated before running on a 3100 Genetic Analyser (Applied Biosystems). The sequencing data was visualized using Sequencher 4.6 (Gene Codes Corporation, Ann Arbor, MI) and was independently analysed by two scientists. Each mutation was confirmed by sequencing a second independent PCR reaction.

Samples were sequenced for *KRAS *exon 2 mutations using an 189 bp amplicon. The sequencing primers were described previously [37]. The reaction mixture in a total of 20 μl was made using HotStarTaq (Qiagen) and contained the following; 1× PCR buffer, 2.5 mM MgCl_2_, 200 nM of each primer, 5 ng of genomic DNA, 200 μM of dNTPs, 0.5 U of Taq polymerase and PCR grade water PCR reactions were performed using the following conditions; initial denaturation at 95°C for 15 min; 50 cycles of 95°C for 10 s, 67.5°C for 10 s, 72°C for 20 s with an initial 10 cycles of touchdown (1°C/cycle); one cycle of 72°C for 10 min. Sequencing reactions were performed as above except that an annealing temperature of 60.7°C and an extension temperature of 72°C were used.

## Results

### EGFR mutation detection by direct sequencing

*EGFR *exons 18 to 21 were sequenced using genomic DNA extracted from FFPE tissues from the 200 NSCLC patients. In total, 73 samples were identified with *EGFR *mutations by sequencing (Table 2). The *EGFR *mutations were most common in exon 19, comprising 61% (46/75) of all mutations found, followed by exon 21 missense mutations in 18% (14/75). Ten mutations in exon 20 (13%) and five mutations in exon 18 (6%) were also detected.

**Table 2:** Summary of *EGFR *mutations detected by sequencing from the 200 NSCLC samples

**Exon**	**Mutation type**	**Nucleotide Change**	**AA change**	**N**
18	missense	c.2126A>C	p.E709A	1
		c.2155G>A *	p.G719S	1
		c.2156G>C	p.G719A	1
		c.2170G>A *	p.G724S	1
	deletion/insertion	c.2127_2130del4insC	p.E709_T710delinsD	1
19	deletion/insertion	c.2233_2245del15	p.K745_D749del	1
		c.2235_2249del15	p.E746_A750del	19
		c.2235_2249del15insTTC	p.E746_A750delinsF	1
		c.2236_2250del15	p.E746_A750del	9
		c.2237_2251del15	p.E746_A750del	1
		c.2237_2252del16insT	p.E746_A750delinsV	2
		c.2237_2238ins18	p.E746VinsPVAIKE	1
		c.2239_2248del10insC	p.L747_D749delinsP	1
		c.2239_2251del13insC	p.L747_T751delinsP	1
		c.2239_2258del20insCA	p.L747_P753delinsQ	1
		c.2240_2254del15	p.L747_T751del	2
		c.2240_2257del18	p.L747_P753delinsS	6
		c.2252_2276del25insA	p.T751_I759delinsN	1
20	missense	c.2303G>T	p.S768I	3
	deletion/insertion	c.2300_2308del9	p.A767_V769del	1
		c.2309_2310insCCAGCGTGG	p.D770_H773insGSVD	1
		c.2311A>G, 2312_2313insGGT	p.N771_P772insGY	1
		c.2317delCinsTACAACCCCT	p.H773_R776insYNPY	1
		c.2322_2323insCCACGT	p.C775_R776insPA	1
	silent mutation	c.2289C>T	p.A763A	1
		c.2313C>T	p.N771N	1
21	missense	c.2506C>T	p.R836C	1
		c.2573T>G	p.L858R	13
	**Total**			**75**

75 mutations were seen in 73 patients. AA: amino acid, N: number of samples.

* These samples showed an additional exon 20 c.2303G>T (p.S768I) mutation.

There were three cases of rare mutations. A 4 bp deletion and 1 bp insertion (p.E709_T710delinsD) and a 9 bp deletion (p.A767_V769del) mutations were detected in exons 18 and 20 respectively. In exon 19, there was a novel in-frame 18 bp insertion mutation that replaced the glutamic acid at p.746 by valine and inserted six amino acids (PVAIKE) afterwards (Table 2).

We also found two samples harbouring double mutations in exons 18 and 20. The mutation in exon 20 in both individuals was the same missense substitution (p.S768I). The exon 18 mutations were p.G719S and p.G724S. The combination of p.G719S and p.S768I mutations has been reported previously in NSCLC samples [38].

Two novel synonymous mutations, p.A763A (c.2289C>T) and p.N771N (c.2313C>T), were found in exon 20. A novel intronic variant with C>T at c.2469+21 was found in a single patient. These may represent passenger mutations or rare SNPs although the germline DNA was not able to be tested to distinguish the two possibilities

### HRM assay with different amounts of DNA template

In many NSCLC cases, the FFPE specimens available for analysis are often small in size. To investigate whether HRM assay can be performed on a small amount of DNA template, DNA amounts ranging from 1 to 100 ng (1, 5, 10, 25, 50 and 100 ng) were tested using a sample with an exon 19 p.E746_A750 deletion mutation present at 50% mutant allele frequency.

**Figure 1:**
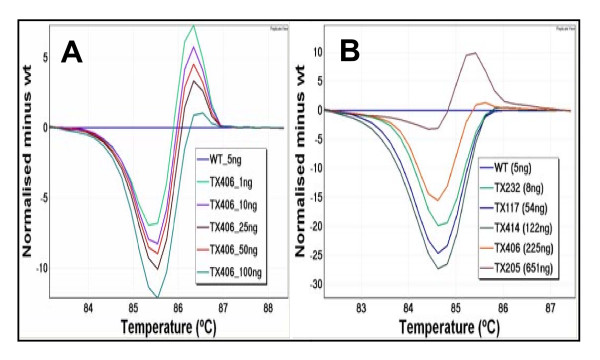
**Difference plots showing mutation detection with various amounts of starting template**. In difference plots, the melting profile of a wild type control is chosen as a horizontal base line and the relative differences in the melting of all the other samples are plotted relative to this baseline. **Panel A**: A difference plot of *EGFR *exon 19 using different amounts of template from one sample. Different amounts of template, ranging from 1 ng to 100 ng, were assessed by the *EGFR *exon 19 assay using a sample with a p.E746_A750 deletion mutation. A near identical heteroduplex type melting pattern is observed from each amount of template tested. The wild-type control sample is shown as a horizontal line (in blue). **Panel B**: A difference plot of *EGFR *exon 19 using different samples with different amounts of template. Each sample contained the same p.E746_A750 in-frame deletion mutation but the samples were at different DNA concentrations and from different individuals. A wild-type control sample is shown as a horizontal line (in blue). All samples show different melting patterns compared to the wild-type.

Distinctive heteroduplex melting patterns were detected across the entire range of DNA template amounts, featuring an earlier melting of amplicon at the initial melting stage and becoming more stable at later stages compared with the wild-type (Figure 1. Panel A). In particular, the melting curve from 1 ng of mutant template was sufficiently different from wild-type to identify the mutation, and this distinct melting profile was consistently seen across all the other template amounts investigated. This illustrates the sensitivity of the technique as mutations can be detected in samples with as little as 1 ng of template DNA by HRM analysis.

After confirming that HRM can be performed with a small amount of template DNA, we further tested the practicality of HRM using a panel of samples with different DNA concentrations, ranging from 8 ng to 651 ng. All samples harboured the same *EGFR *exon 19 p.E746_A750 deletion mutation. Again, all mutant samples exhibited distinct melting characteristics from wild-type, and could easily be identified as mutants (Figure 1. Panel B).

These analyses demonstrated that reliable results could also be obtained when more than 1 ng of template DNA was tested by HRM. For the samples that amplified well, the variation in the amount of template within samples had minimal influence on the HRM analysis, indicating that adjusting the amount of template might normally be unnecessary.

### *EGFR and KRAS *mutation detection by HRM analysis

**Figure 2:**
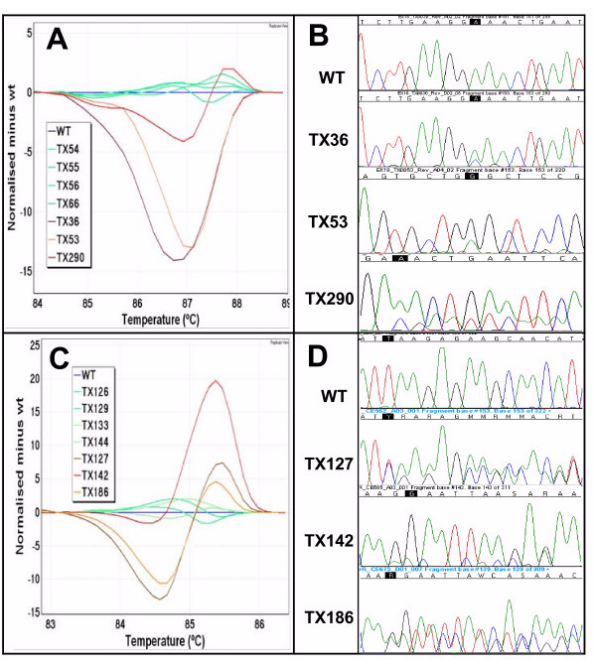
**Difference plots and sequence traces for *EGFR *exons 18 and 19**. **Panel A**: The difference plot of *EGFR *exon 18 shows melting profiles for three positive samples (TX36 in purple, TX53 in orange and TX290 in red). **Panel B**: Sequencing chromatograms show a p.E709A in TX36, a p.G719S in TX53 and a p.E709_T710delinsD in TX290. **Panel C**: The difference plot of EGFR exon 19 shows melting profiles for three positive samples (TX127 in brown, TX142 in red and TX186 in orange). **Panel D**: Sequencing chromatograms show a p.L747_P753delinsS in TX127, a p.E746_A750del in TX142 and a p.E746_A750delinsF mutation in TX186.

**Figure 3:**
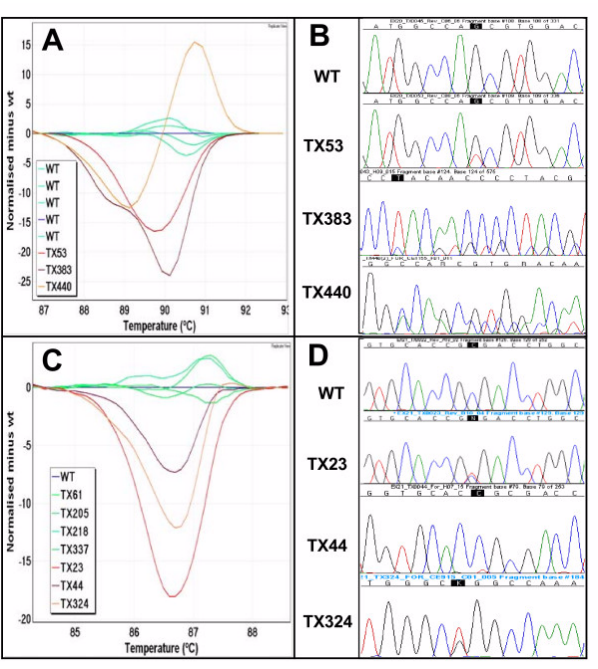
**Difference plots and sequence traces for *EGFR *exons 20 and 21**. **Panel A**: The difference plot of *EGFR *HRM exon 20a shows different melting profiles of three positive samples (TX53 in red, TX383 in brown and TX440 in orange). **Panel B**: A p.S768I in TX53, a p.H773_R776insYNPY in TX383 and a p.D770_H773insGSVD in TX440 mutation are detected by sequencing from the HRM positive samples. **Panel C**: The difference plot of *EGFR *HRM exon 21 shows three HRM positive samples (TX23 in red, TX44 in purple and TX324 in orange). **Panel D**: Sequencing chromatograms show a p.R836R SNP in TX23, a p.R836C in TX44 and a p.L858R mutation in TX324.

All 200 NSCLC samples were tested by HRM for the detection of mutations in *EGFR *exons 18 to 21 and *KRAS *exon 2. The *EGFR *HRM assays gave 23, 67, 23 and 41 results that were scored as HRM positive in exons 18 to 21 respectively (Table 3). All mutations identified by sequencing were correctly identified by HRM assays giving a sensitivity of 100%. The difference plots for *EGFR *exons 18 to 21 and sequencing traces of positive samples are shown in Figures 2 and 3.

**Table 3:** Summary of *EGFR *mutation testing by sequencing and HRM

**Exon**	**SEQ Positive**	**HRM Positive**	**HRM positive only**	**Sensitivity (%)**	**Specificity (%)**
18	5	23	18	100	91
19	46	67	21	100	88
20	10	23	13	100	93
21	23*	41	18	100	91

* The nine samples containing the SNP c.2508C>T were included.

SEQ: sequencing, HRM: high resolution melting.

However, HRM indicated more positive samples than sequencing for all of the *EGFR *exons. A total of 45 samples were positive only by HRM. There were 18, 21, 13 and 18 apparently false positive results from exons 18 to 21 respectively. Significantly, seven samples were positive in three or four *EGFR *HRM assays, and eight samples were positive in two assays. Most of these are likely to be true false positives due to degraded DNA from the FFPE specimen. Thirty samples were positive in a single assay only. Compared to other assays, the number of samples with discrepant results was lower in the exon 20 assays where relatively shorter amplicons were tested, further highlighting the importance of using shorter HRM amplicons to enhance the reliability of the results.

**Figure 4:**
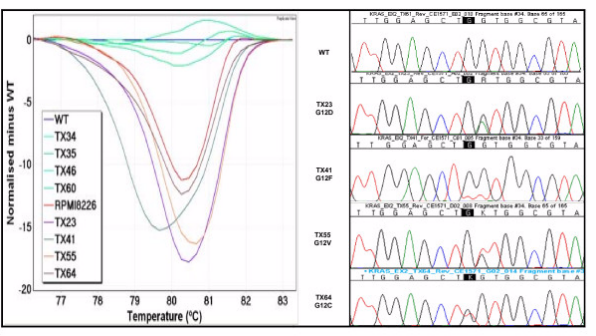
**Difference plots and sequence traces for *KRAS *exon 2 mutations**. In the difference plot for *KRAS *exon 2, the melting curves from the four samples (TX23 in purple, TX41 in green, TX55 in orange and TX64 in brown) and a positive mutation control (RPMI8226 in red) were compared to a wild-type sample. The wild-type control sample is shown as a horizontal line in blue. Four patients and the positive control samples had different melting profiles compared to the wild-type. Sequencing of the patients revealed four different amino acid changes at codon 12; p.G12D in TX23, p.G12F in TX41, p.G12V in TX55 and p.G12C in TX64.

*KRAS *exon 2 mutations at codons 12 to 13 were screened by our previously described HRM assay [28] that generated a 92 bp PCR amplicon (Figure 4). Twenty-five samples of the 200 tested (12.5%) were scored as HRM positive. These samples were negative for *EGFR *mutations by both sequencing and HRM.

All 25 *KRAS *exon 2 HRM positive samples proved to have a mutation at codons 12 or 13 when tested by sequencing (Table 4). Seven different *KRAS *mutations were observed amongst the 25 mutation positive samples, comprising 5 mutations at codon 12 (8 p.G12C, 7 p.G12D, 5 p.G12V, 1 p.G12F and 1 p.G12A) and 2 mutations at codon 13 (2 p.G13C and 1 p.G13V). In addition, 25 samples that were both *EGFR *mutation negative and *KRAS *mutation negative were sequenced for *KRAS *exon 2. All showed normal *KRAS *sequence.

**Table 4:** Summary of *KRAS *exon 2 mutations detected by HRM and sequencing

**Mutation**	**AA change**	**N**
c.34G>T	p.G12C	8
c.34_35GG>TT	p.G12F	1
c.35G>A	p.G12D	7
c.35G>T	p.G12V	5
c.35G>C	p.G12A	1
c.37G>T	p.G13C	2
c.38_39C>TT	p.G13V	1
**Total**		**25**

AA: amino acid, N: number of samples

## Discussion

The advent of molecularly targeted therapy is shifting the paradigm of management of cancer patients from generalised chemotherapy and/or radiotherapy to personalised treatments with better efficacy and lower toxicity. An example of this is the development of *EGFR *tyrosine kinase inhibitors in the management of NSCLC patients where the presence of predictive markers such as *EGFR *and *KRAS *mutations in their tumours stratifies patients to receive the appropriate treatment.

Currently, direct sequencing is the standard method for EGFR mutation detection, but its limited sensitivity, high cost and long turnaround time have prompted the development of alternative methods for routine clinical testing which have greater diagnostic practicality for somatic mutation detection.

HRM has recently been introduced as a screening method for mutation detection. It is an in-tube method which can be performed in a fast, cheap, and robust manner. It has been applied to the detection of both germline and somatic mutations. For heterozygous germline mutations, HRM has sensitivities approaching 100% [32,39]. For somatic mutations in tumours, detection can be compromised by a low proportion of tumour cells in the biopsy. In practice, it has been shown previously that mutant alleles at levels as low as 5 to 10% can be detected by HRM for *KRAS *exon 2 mutations [29,37].

HRM has previously been used for detection of *EGFR *mutations. In lung cancer, the two most common types of *EGFR *mutation, exon 19 deletions and exon 21 p.L858R, were screened by HRM, with a reported 92% sensitivity compared with direct sequencing [29,40]. In head and neck cancer, *EGFR *exons 18 to 21 were screened by HRM, resulting in the detection of two *EGFR *mutations in 24 squamous cell carcinomas [28].

We designed this study to determine whether HRM can be a diagnostically useful screening method for *EGFR *and *KRAS *mutations in clinical FFPE specimens by validating HRM against sequencing in a large sample cohort containing various types of *EGFR *and *KRAS *mutations.

Each of the previously described HRM mutation screening assays for *EGFR *have limitations preventing them from being practical for one or more of the following reasons: they do not cover all of exons 18 to 21 [29,40], they do not discriminate against common SNPs leading to unnecessary sequencing [28], they do not detect all mutations that are detectable by sequencing [29,40] and they have not been validated against a large panel of clinical samples with previously determined mutations [28].

In one assay, a primer is located over the c.2184+19 common SNP potentially resulting in non-amplification of the mutant allele [28]. Allele dropout due to sequence variation at the primer binding site has been previously demonstrated [41]. If the allele that has not amplified contains the mutation, the mutation will not be detected.

In this study, HRM assays were designed to maximise the benefits of HRM screening and to minimise SNP interference. *EGFR *exon 18 to 21 assays covering the entire coding regions were developed. The incorporation of mismatched bases at SNP loci within the primer sequences made it possible to exclude two SNPs, c.2361G>A and c.2184+19G>A. Under the conditions used in our assays, this did not preclude efficient amplification of both alleles, allowing us to detect all 15 mutations from exons 18 and 20.

Due to its high frequency, the exonic SNP, c.2361G>A, would normally necessitate nearly 50% of samples being sequenced for exon 20 because the heterozygous melting pattern given by the SNP can not readily be distinguished from mutation by HRM. Fortunately, its position, in the middle of a large exon, allowed us to divide exon 20 into two fragments using two overlapping amplicons with PCR product sizes of 121 bp and 146 bp. Primers which overlaid the SNP and contained a mismatched residue at the SNP location were used for both amplicons to exclude the SNP from being detected by HRM. A 'G' and an 'A' respectively (mismatched to both alleles) were introduced at c.2361 into the exon 20a reverse and exon 20b forward primers. This strategy led to a short region of 15 bp (c.2353_2367) flanking the SNP for which mutations could not be detected. This is not a serious limitation as no mutations have so far been reported in that region [42].

The other common exonic SNP, c.2508C>T in exon 21, is present at a much lower frequency and thus necessitated only a comparatively small increase in the amount of sequencing that would be required. The SNP was detected in nine of the 200 samples.

**Figure 5:**
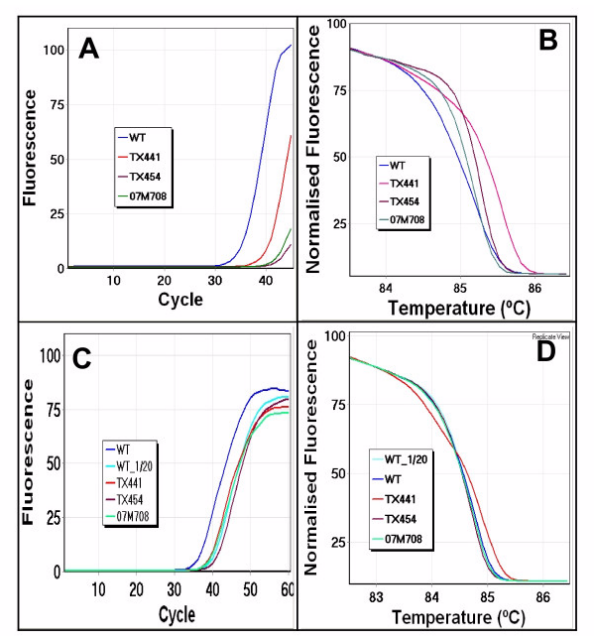
**Influence of amplification status on melting analysis**. **Panel A: **The amplification plot of *EGFR *exon 19 shows insufficient amplification of several samples relative to the wild-type (blue). **Panel B: **The subsequent melting analysis of the same samples on a normalised plot shows the right shift of curves from the wild-type. **Panel C: **Dilution (1 in 20) of the wild-type (aqua) and the addition of 15 cycles allowed all samples to achieve similar levels of amplification. **Panel D: **The normalised plot now shows no aberrant right shifting of melting curves, identifying TX441 as mutation positive and TX454 and 07M708 as mutation negative.

It is important to consider the amplification information when interpreting HRM results. Instruments allowing the real time monitoring of amplification are advantageous in this regard. We observed that melting curves from samples with insufficient amplification tended to be shifted to the right relative to the wild-type curves in the normalised plots. This implies that the amount of the amplifiable (functional) templates varies in each sample depending on the degree of DNA degradation even though they are all adjusted to the same concentration (2.5 ng/μl). For those samples, we diluted the wild-type control DNA to get similar amplification to the samples with insufficient amplification. We also increased the amplification cycle number to 60 to allow sufficient amplification for melting analysis. As shown in Figure 5, with sufficient amplification, the right shifting of melting curves was corrected and thus the patient DNA could be reliably compared to the wild-type.

Accurate identification of mutations is a crucial aspect of all mutation screening methods. This current study demonstrates the accuracy of HRM in the detection of *EGFR *and *KRAS *mutations in a panel of 200 NSCLC samples. Seventy-five *EGFR *mutations and 25 *KRAS *mutations were identified by HRM analysis in concordance with sequencing results. The mutation types for each exon were similar to those reported in previous studies, in-frame deletions in exon 19, insertions in exon 20 and missense mutations in both exon 18 and 21 [7-9]. However, the overall mutation rate (37.5%) in this Australian study was much higher than in North America and Western Europe (10%) but similar to East Asian populations (30–50%) [43-47]. Although most Australians are of European descent, the higher *EGFR *mutation rate is likely to be due to selection bias of patients by referring oncologists for features associated with EGFR mutation. The pre-selection criteria included tumours that were histologically adenocarcinomas (70% of samples tested) and patients that were female (60% of patients tested) and/or of Asian ethnicity as many of the patients had a name that was consistent with Asian ancestry. The *KRAS *mutation rate (12.5%) which is lower than that reported in previous studies [16,17] also supports the notion of pre-selection bias.

Although all positive sequencing results were detected by HRM, some samples were considered positive by HRM but were negative by sequencing (Table 3). There are several possible explanations. One possible explanation is that the adverse effects of formalin fixation on DNA can cause PCR artefacts during amplification. At least four chemical reactions occur between formaldehyde and DNA; methylol formation, methylene bridge formation, apurinic and apyrimidinic site formation, and hydrolysis of the phosphodiester bonds [48]. Compared to the DNA extracted from frozen tissues, a higher frequency of non-reproducible sequence alterations have been reported with DNA isolated from the formalin fixed tissues [49,50]. Therefore, the cumulative effects of the PCR artefacts either from Taq polymerase error and errors attributed from chemical reactions of formalin on DNA influence the melting profile of the amplicon depending on the degree of DNA damages.

Seven samples gave positive results in more than three *EGFR *HRM assays, supporting the hypothesis that the quality of DNA is one of the factors causing aberrant variation of melting in HRM analysis. It has been observed that the wild-type variation in melting analysis is much greater with FFPE DNA than DNA from frozen tissues or peripheral blood (data not shown). The amount of false positives decreased with decreasing amplicon length, with *EGFR *exon 19 (amplicon size 250 bp) giving the most false positives and *KRAS *exon 2 (92 bp) giving the least.

Another possibility is that some samples contained levels of mutation below the sensitivity of sequencing detection as a result of a low percentage of tumour in the sample or genetic heterogeneity within the tumour. Where sequencing requires the mutation to be present at a level of 20% of the sample, HRM can detect heterozygous genetic changes down to 10% or below [26,29,37]. We are now adopting digital techniques to confirm that some specimens have true mutations present at low levels. Other HRM false positives can arise from PCR errors due to amplification from very low levels of template. True mutations can be distinguished from artefacts by confirming the identical sequence variations from independent amplification (Do and Dobrovic, manuscript submitted).

HRM is a suitable methodology to test FFPE samples as well as samples with a very low quantity of DNA. It has been reported that ten percent buffered formalin, an aqueous dilution of formaldehyde, can interact with DNA and initiate irreversible DNA degradation resulting in an adverse effect on DNA quality [35]. In our HRM assays, all the samples were successfully amplified and analysed. Our results show that HRM can be performed with as little as 1 ng template in the *EGFR *exon 19 HRM assay. This level of sensitivity of HRM, together with the possibility of sequencing of the HRM product, will extend our ability to screen even clinical samples with extremely low DNA quantity such as samples taken from patients with inoperable tumours. HRM analysis can now provide a genetic testing option for these patients, in which the results might prove useful in directing treatment and may ultimately improve outcomes.

*EGFR *and *KRAS *mutations are predominantly mutually exclusive with very rare tumours containing both genes mutated [44,51]. The coexistence of mutations in both *EGFR *and *KRAS *has only been reported in two patients [52]. In the current study, all 25 *KRAS *positive samples were wild-type for *EGFR*, supporting the general mutual exclusiveness of the two mutations.

## Conclusion

We have established a fast, efficient and reproducible screening method for *EGFR *and *KRAS *mutation detection by which degraded DNA from FFPE tissues can be tested. This is the first study in which the HRM method has been properly validated as a scanning method for *EGFR *and *KRAS *mutations in a large sample set showing accurate mutation detection with 100% sensitivity. It is estimated that up to 80% of sequencing reactions can be eliminated for these two genes if samples are screened by HRM. These results further demonstrate the utility of HRM for the detection of somatic mutations in clinical samples and for screening of samples prior to sequencing.

## Competing interests

The authors declare that they have no competing interests.

## Authors' contributions

HD participated in the development of the assays carried out the HRM studies, prepared the figures and wrote the manuscript. MK assisted in development of the assays and in data analysis. SBF contributed to the manuscript and provided access to the sequencing data and to the specimens. This study was initiated as part of a grant to PLM and AD. AD initiated the study, was responsible for primer design, participated in the data analysis, and co-wrote the manuscript. All authors read and approved the final manuscript.

## Pre-publication history

The pre-publication history for this paper can be accessed here:


